# Technical Feasibility of IVIM for Prostate Cancer Imaging Using High‐Amplitude Whole‐Body Gradient System With Spiral Readout and Velocity‐Compensated Diffusion Encoding

**DOI:** 10.1002/nbm.70384

**Published:** 2026-09-09

**Authors:** Malwina Molendowska, Ivan A. Rashid, Kieran Foley, Lars Mueller, Lars E. Olsson, Patrik Brynolfsson, Filip Szczepankiewicz

**Affiliations:** ^1^ Medical Radiation Physics Lund University Lund Sweden; ^2^ Cardiff University Brain Research Imaging Centre (CUBRIC) Cardiff University Cardiff UK; ^3^ Department of Translational Medicine, Medical Radiation Physics Lund University Malmo Sweden; ^4^ Division of Cancer and Genetics, School of Medicine Cardiff University Cardiff UK; ^5^ Leeds Institute of Cardiovascular and Metabolic Medicine University of Leeds Leeds UK; ^6^ Radiation Physics, Department of Hematology, Oncology and Radiation Physics Skane University Hospital Lund Sweden

**Keywords:** IVIM, perfusion, prostate cancer, strong gradients, velocity compensation

## Abstract

Intravoxel incoherent motion (IVIM) is sensitive to tissue diffusion and perfusion and has spurred great interest in various pathologies such as prostate cancer. However, poor repeatability and reproducibility of IVIM parameters hamper its wide‐scale adoption in the clinics. To address this, improvements to the IVIM data acquisition and analysis are necessary. The purpose of this study was to develop a framework for high‐quality IVIM imaging of the prostate. To boost signal precision and quality of IVIM parameter maps, we used a custom pulse sequence to combine velocity‐compensated diffusion encoding with spiral readout at a high‐performance 3 T MRI system with 300 mT/m gradients. IVIM parameters were evaluated in a cohort of five men with and without prostate cancer. Technical performance was assessed in terms of the signal‐to‐noise ratio (SNR), visual quality of IVIM parameter maps and parameter repeatability. High in‐plane resolution images of the prostate were obtained with SNR > 3 (i.e., noise floor level) in 91% of voxels at the highest b‐value of 500 s/mm^2^. The parameter maps exhibited high quality with fine features recovered in tissue diffusivity and perfusion fraction maps. Voxel‐wise and ROI‐based analysis revealed high repeatability of the parameters at single subject and group level across main prostatic tissue classes (peripheral and transitional zones) and prostate cancer. A combination of velocity‐compensated diffusion encoding, spiral readout and strong gradients enables high‐quality IVIM imaging in the prostate. Our framework may advance IVIM as a reliable imaging biomarker in prostate.

## Graphical abstract

The combination of velocity‐compensated diffusion encoding, spiral readout and strong gradients enabled high‐quality prostate IVIM imaging, offering a promising framework for the development of reliable IVIM‐based biomarkers for prostate cancer detection, characterisation and monitoring.

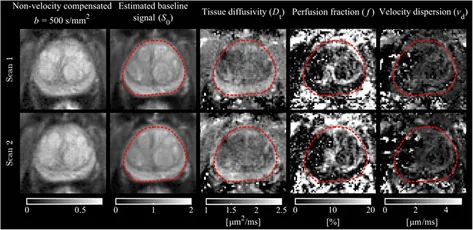

## 1 Introduction

Intravoxel incoherent motion (IVIM) is a diffusion MRI (dMRI) method that enables mapping of tissue microstructure and perfusion, without relying on exogenous contrast agents [1]. The potential of IVIM has been demonstrated across various organs and pathologies [2, 3, 4, 5], resulting in promising candidates for imaging biomarkers, e.g., for the diagnosis of prostate cancer and its monitoring [6, 7]. However, the estimation of IVIM parameters is challenging due to ill‐posedness, which places a relatively high demand on data quality. As a result, several studies have shown unreliable parameter estimation and contradictory findings [8].

These challenges are further exacerbated in prostate imaging, which suffers from relatively low signal precision due to its small size requiring high spatial resolution, its distance from receiver coils, involuntary motion in the bladder, rectum and bowels, and non‐stationary distortions caused by moving gas [9]. Conventional prostate dMRI employs a single‐shot spin echo with EPI readout, where diffusion encoding is performed with monopolar trapezoidal gradients [10, 11]. However, it has two major drawbacks. First, such diffusion encoding does not control the encoding of ballistic motion, which can be used to distinguish incoherent blood flow from diffusion. Second, the EPI readout starts before the echo time, which reduces the time available for diffusion encoding.

In these challenging circumstances, the impact of poor data quality can be partially mitigated at the analysis stage. To this end, several methods for parameter estimation have been proposed, including segmented [12], Bayesian [13, 14] and deep learning–based [15] approaches. Generally, these approaches achieve improved parameter precision and robustness by implicit or explicit regularisation. The drawback of regularisation is that it may also introduce a parameter bias. Recent efforts have been made to standardise IVIM as part of a consensus [16]. One guideline argues that simpler fitting algorithms may be preferable to enable straightforward implementation and enhanced reproducibility, although it also places greater demands on the quality of the underlying data. Recently, several advances have been proposed to improve both the diffusion encoding and image readout, which can be leveraged in IVIM imaging. The addition of velocity‐compensated diffusion encoding introduced a means by which the contribution from ballistic flow could be modulated [17]. This modification yielded more robust parameter estimation, with promising demonstrations in the brain, liver and kidney [17, 18, 19]. In a similar fashion, the overall quality of MR images can be enhanced through more efficient signal readout strategies, such as spiral readout trajectories [20]. Spirals can start close to TE yet still sample the full k‐space, thereby maximising the time available for diffusion encoding [21] and/or reducing the TE to improve SNR [22]. Taken together, these developments indicate that accuracy of microstructure parameters can be improved by careful experimental design and advances in MRI hardware. This has arguably motivated several MRI vendors to design systems with strong gradients [23, 24, 25], with tangible improvement across a wide range of applications [26, 27, 28]. Thus, a combination of tailored diffusion encoding, enhanced image readout and high‐amplitude gradients can be leveraged to improve IVIM.

In this work, we propose and demonstrate a framework for state‐of‐the‐art IVIM imaging of the prostate. To achieve high‐quality data and parameter maps, we leverage the combined benefits of the diffusion encoding with variable velocity‐encoding, high‐amplitude gradients, spiral image readout and field monitoring for image reconstruction. We deployed this framework in a group of prostate cancer patients and characterised technical performance in terms of the signal‐to‐noise ratio (SNR), visual quality of IVIM parameter maps and parameter repeatability.

## 2 Materials and Methods

### 2.1 IVIM

Proposed by Le Bihan et al. [1], IVIM describes the low b‐value diffusion‐weighted signal as a combination of the diffusion of water in tissue, and the motion of blood in capillary vessels, or pseudo‐diffusion, which includes both diffusion and incoherent flow. Conventionally, by sampling a range of low‐to‐intermediate b‐values, these two components are separated: at low b‐values, the signal originates from both blood and tissue, whereas at higher b‐values, the signal from blood is strongly suppressed, which isolates the signal from tissue. Since IVIM experiments are sensitive to the blood in capillary vessels as part of microcirculation, the signal dynamics can be linked to perfusion [29].

Depending on the time scale of the diffusion encoding, the signal from blood reflects on two distinct mechanisms [29]. At relatively short encoding times, spins tend to stay in straight capillary segments and maintain constant velocities, characteristic of the ballistic limit. At longer encoding times, the spins have time to traverse multiple segments and experience multiple changes in velocity, resulting in pseudo‐random trajectories, characteristic of the diffusive limit.

The signal that is attenuated due to directionally incoherent ballistic motion can be recovered using velocity compensation. A joint analysis of diffusion encoding with and without velocity compensation therefore allows separation of ballistic and diffusive effects [30, 31], and can thereby improve the stability of parameter estimation [17, 18]. In the ballistic regime, the diffusion and velocity‐encoded signal is [17]:

(1) Sbm1S0=1−fe−bDt+fe−bDb+m12vd2

where *S*_0_ is the signal without diffusion weighting, *b* is the diffusion encoding strength, *f* and (1 − *f*) are the perfusion and tissue signal fractions (0 ≤ *f* ≤ 1), *D*_b_ and *D*_t_ are apparent diffusion coefficients in blood and tissue, respectively, and *v*_d_ is the velocity dispersion of the blood flow. The magnitude of velocity encoding, *m*_1_, is the norm of the first moment of the effective gradient waveform, **g**(*t*), according to [32]:

(2) $$m_{1}={\left\|\gamma \int_{\;0}^{\mathrm{TE}}g\left(t\right)t\;dt\right\|}_{2}$$

where *t* is the time, TE is the echo time and γ is the gyromagnetic ratio. Velocity compensation implies that diffusion encoding gradient waveform, **g**(*t*), has a negligible first gradient moment *m*_1_, while non–velocity‐compensated gradients have a non‐zero first moment (*m*_1_ > 0). For all spins that move at constant velocities during the experiment, velocity compensation nulls the phase shift caused by the encoding gradients, regardless of the distribution of velocities. If *m*_1_ is not zero, the average motion of spins will cause a phase shift, and any incoherence in velocities (e.g., due to different directions in the capillaries) will cause a dephasing, which is pseudo‐random and therefore similar to diffusion [17].

### 2.2 Gradient Waveform Design

#### 2.2.1 Velocity‐Compensated Diffusion Encoding

**FIGURE 1:**
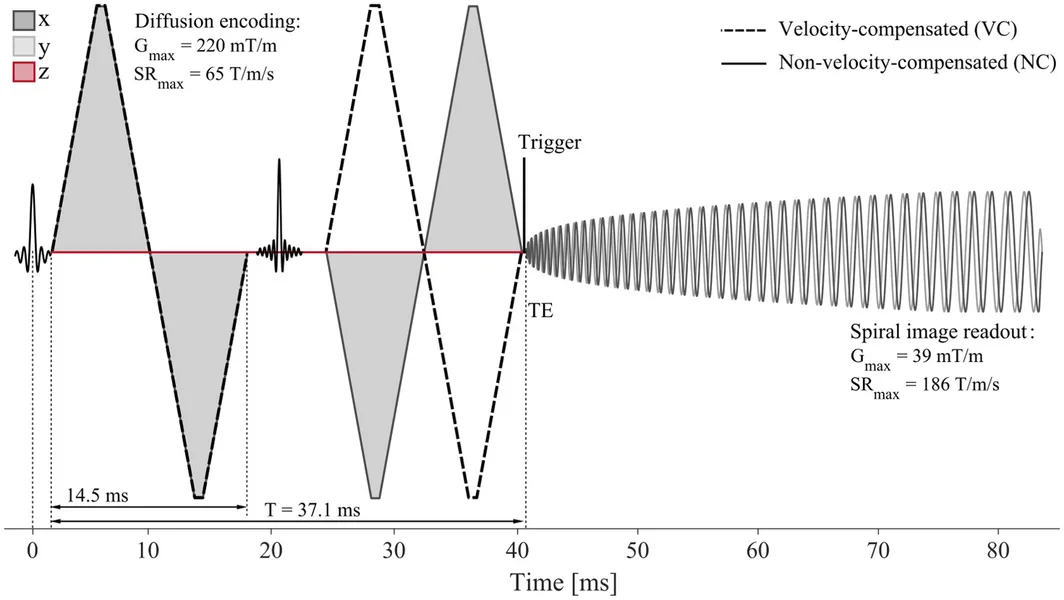
Spin‐echo pulse sequence diagram shows bipolar diffusion encoding waveforms that are compensated and non‐compensated for velocity (VC vs. NC) and single‐shot spiral trajectory for image encoding. G_max_ and SR_max_ are maximal gradient amplitude and slew rate used, and T is the total diffusion encoding time. The solid black line shows the trigger that engages the field camera recording. Please refer to the Materials and Methods section for key experimental parameters.

Gradient waveforms for diffusion encoding were designed to be non‐compensated (NC) or velocity‐compensated (VC) by using a (anti‐)symmetric bipolar waveform [33] with varying polarity after the refocusing pulse (Figure 1). The duration of each bipolar block was 14.5 ms with a separation gap of 8.1 ms; the total diffusion encoding time was 37.1 ms. The gradient waveforms were scaled to achieve nine nominal encoding strengths, *b* = [10, 20, 30, 50, 100, 150, 200, 300, 500] s/mm^2^ executed along six directions. Thirteen acquisitions without diffusion encoding were acquired. The order of acquisitions was randomised to minimise system drift effects [34]. The nominal values of *m*_1_^2^/*b* for compensated and non‐compensated encoding were 0.0 and 18.9 ms. The slew rate of gradient ramps was set to 65 T/m/s, and the maximum gradient amplitude was set to 220 mT/m to maintain acceptable levels of peripheral nerve stimulation (PNS). The norm of the PNS, estimated at the highest b‐value and the worst waveform rotation, was 83% of the allowed maximum (https://github.com/filip‐szczepankiewicz/safe_pns_prediction).

#### 2.2.2 Spiral Image Readout Trajectory

The 2D spiral readout (Figure 1) was designed using the ‘time‐optimal gradient design’ toolbox [35] for FOV = 220 × 220 mm^2^ with in‐plane spatial resolution = 1.15 × 1.15 mm^2^, and an undersampling factor of 2.24, yielding readout duration of 44 ms with maximum slew rate and gradient amplitude of 186 T/m/s and 39 mT/m. The norm of the PNS for the spiral readout gradients (executed in axial plane, x–y physical gradient axes) was 96%.

### 2.3 In Vivo Imaging of Prostate Cancer Patients

Data was obtained with 3 T MRI MAGNETOM Connectom research‐only system with 300 mT/m gradient coil, using an 18‐channel body coil and a 32‐channel spine coil for signal reception (Siemens Healthineers, Forchheim, Germany).

IVIM diffusion‐weighted imaging (DWI) was acquired using a slice selective single‐shot spin echo sequence with diffusion weighting and spiral image readout (see ‘Gradient Waveform Design’) [36]. The acquisitions were performed in two series, with either velocity‐ or non–velocity‐compensated gradients. The remaining acquisition parameters were TE = 41 ms, TR = 3 s, slice thickness = 5 mm, number of slices = 18, no slice gap, CHESS fat suppression, interleaved slice acquisition, scan time = 7:00 min.

Multi‐echo gradient echo images were acquired to map the static off‐resonance and receiver coil sensitivities. The imaging parameters were: in‐plane FOV = 440 × 260 mm^2^, in‐plane spatial resolution = 3.07 × 3.07 mm^2^, slice thickness = 5.0 mm, TE = [2.32, 4.64, 6.96, 9.28] ms, TR = 547 ms, number of slices = 18, no slice gap, scan time = 2:00 min.

Following clinical guidelines [37], conventional (‘clinical‐like’) DWI was acquired using the vendor's product sequence and reconstruction, using the available gradient amplitude for a maximum b‐value of 800 s/mm^2^. Imaging parameters were: FOV = 260 × 260 mm^2^, in‐plane spatial resolution = 1.33 × 1.33 mm^2^, slice thickness = 5 mm, TE = 59 ms, TR = 3 s, no slice gap, CHESS fat suppression, interleaved slice acquisition, scan time = 2:27 min.

Morphological imaging was performed using a 2D T_2_‐weighted turbo spin echo sequence in axial plane with TE = 97 ms, TR = 4 s, in‐plane spatial resolution = 0.63 × 0.63 mm^2^, slice thickness = 3 mm, slices = 23, no slice gap, in‐plane FOV = 200 × 200 mm^2^, PE = right–left. The total acquisition time of the structural scan was 3: 20 min.

### 2.4 Patient Cohort and Scanning Schedule

The data was acquired from five men either enrolled in ‘active surveillance’ or previously screened for prostate cancer with a negative biopsy. All subjects were recruited through the National Health Service (NHS) in Wales, UK. The participants were advised to follow a low‐residue diet for 24 h prior to imaging. The study was performed in accordance with ethical approval (NHS Wales, REC reference: 21/EM/0284).

The patient demographic details were (mean value ± standard deviation [min, max]): age = 69 ± 3 [66, 73] years, weight = 84 ± 9 [71, 95] kg, height = 1.74 ± 0.04 [1.68, 1.78] m, with biopsy‐confirmed prostate cancers (Gleason score: 3 + 3 or 3 + 4).

For test–retest analysis, three patients had repeated IVIM scans within session (no repositioning), and two patients had scans on separate sessions. Detailed patient information is given in Table 1.

**TABLE 1:** Patient cohort information.

ID	Age (years)	Height (m)	Weight (kg)	Diagnosis (Gleason score)	Tissue volume (cm^3^) whole prostate/peripheral/transitional/prostate cancer	Time between scans
1	66	1.72	71	3 + 4	28.5/10.6/16.5/0.7	17 days
2	69	1.78	90	3 + 3	31.2/10.9/16.7/2.2	91 days
3	67	1.68	83	3 + 3	53.5/9.2/42.6/0.05	0 days (21 min)
4	71	1.77	83	3 + 3	32.2/15.0/14.0/2.0	
5	73	1.77	95	—	36.9/11.1/24.3/—	

*Note:* A whole prostate volume consists of peripheral, transitional and central zones. We did not exclude benign prostatic hyperplasia nodules from our analysis and we excluded anterior fibromuscular stroma and urethra tissue classes.

### 2.5 Reconstruction of Diffusion‐Weighted Images

For the reconstruction of diffusion‐weighted MRI data, field dynamics during the readout were monitored in a sequential experiment, using a dynamic field‐camera (Skope Magnetic Resonance Technologies AG, Zurich, Switzerland) [38].

We used a dedicated image reconstruction method called the expanded encoding model to correct for field imperfection using the recordings of field evolution from field monitoring experiment [39]. For further details please refer to previous works [26, 40].

### 2.6 Data Processing and IVIM Parameter Estimation

Real‐valued images were recovered from complex image data using EDDEN [41] and denoised using a Marchenko–Pastur principal component analysis (MPPCA) [42, 43], followed by motion correction with Elastix (version 4.9, [44]) with affine transformations to account for deformations of the prostate.

IVIM parameters were estimated by fitting Equation (1) to the signal. The implementation used a non‐linear least‐squares algorithm from scipy (version 1.14.1) with a two‐step fitting approach. First, the fitting was initialised with a random guess from uniform distributions within the parameter bounds. The second fit was initialised with a guess that was based on the local (3 × 3 pixels) parameter median. This approach reduced the number of outliers caused by poor initial guesses without imposing any regularisation or limiting the space of possible solutions. For both fitting steps, the same bounds were used: *f* ∈ [0, 50] %, *D*_t_ ∈ [0, 4] μm^2^/ms and *v*_d_ ∈ [0, 5] μm/ms with *D*_b_ set to a constant value 1.54 μm^2^/ms [45]. The *soft_l1* loss function was used together with tolerances *f*_tol_ = 0.05, g_tol_ = 1e‐8, x_tol_ = 1e‐8 and f_scale_ = 1. Finally, to improve the accuracy of the estimated parameters [46, 47], we used the entire gradient waveform, including imaging gradients to calculate the actual b‐value according to the following equations:

(3) $$b=\text{Trace}\left(B\right)$$

where

(4) $$B=\int_{\;0}^{\mathrm{TE}}q\left(t\right)⊗q\left(t\right)dt$$

and

(5) $$q\left(t\right)=\gamma \int_{\;0}^{t}g\left(t^{\prime }\right)dt^{\prime }$$

where ⊗ denotes the vector outer product. Similarly, the actual velocity encoding was calculated according to Equation (2). The precision and accuracy of the two‐step fitting approach against the single‐step variant is presented in the Supplementary Materials.

Region of interests (ROI) in tumours were manually delineated by an experienced radiologist (6 years of experience). The healthy‐appearing tissue in peripheral, transitional and central zones was defined using automatic segmentation of T_2_‐weighted images [48]. Using the segmented tissue classes and ROIs in cancerous lesions, approximate tissue volumes in cm^3^ were calculated. The volumes are reported alongside patient information in Table 1.

### 2.7 Analysis of Signal to Noise Ratio

The rectified noise floor introduces positive bias in MRI signal affecting accuracy of parameters [49, 50]. To investigate the potential impact of low SNR, we calculated the voxel‐wise SNR at each b‐value according to SNR(*b*) = *E*(*S*(*b*))/std(*S*_0_), where *E* is the expected value (mean) and std is the standard deviation. We estimated the standard deviation of signal from the repetitions at *b* = 0 s/mm^2^, and the signal was the average across rotations and encoding type at a given b‐value. To avoid overestimation, the SNR was estimated using the magnitude of the complex data prior to data processing. Assuming the signal exhibits an approximate Rician distribution, we emphasised regions with SNR < 3, where Rician bias compromises signal accuracy [51]. Distributions of voxel‐wise SNR within the whole prostate gland were pooled across subjects and sessions and compared across b‐values. The whole‐gland masks were defined as the combined ROIs of the segmented tissue classes.

### 2.8 Analysis of Repeatability

For within‐sessions voxel‐wise analysis, we calculated differences (*ΔX* = *X*_1_ − *X*_2_) between the first and second acquisitions [52], yielding *ΔX* as a distribution of voxel‐wise differences. The distribution of *ΔX* was visualised using a single slice from a representative subject as well as Bland–Altman plots. The repeatability was summarised by the mean and standard deviation of *ΔX*. For Bland–Altman analysis, we defined either a slice or whole volume prostate gland mask (comprising cancerous lesions and peripheral, central and transitional zones) and refined them by excluding voxels where *f* was <0.5%, to avoid wrongful contributions from negligible subcomponents (*f* and *v*_d_). To reduce variability from slight inter‐session misalignments, the tissue segmentations were transferred to respective scan or rescan diffusion‐weighted data space, and only the spatial overlap across both scans was considered in the analysis. Finally, we assessed the values of IVIM parameters at single‐subject and group level within the three tissue classes (peripheral and transitional zones, and prostate cancer) by evaluating the mean and standard deviation of the values for scan/rescan session separately.

## 3 Results

The patients involved in the study completed the experiment and did not report any adverse effects due to a scan with strong gradients or its duration (7 min). Regarding the experimental design, the maximal deviation of actual b‐ and m_1_‐values from nominal ones was approximately 1% (Table 2).

**TABLE 2:** The nominal and actual experimental variables *b*, *m*_1_ and *m*_1_/*b* for the non–flow‐compensated diffusion waveforms.

Nominal *b* (s/mm^2^)	Actual *b* (s/mm^2^)	Nominal *m*_1_ (s/mm)	Actual *m*_1_ (s/mm)	Actual *m*_1_^2^/*b* (ms)
0.0	0.2	0.00	0.03	0.0
10.0	10.3	0.43	0.45	20.4
20.0	20.4	0.61	0.64	19.6
30.0	30.5	0.75	0.77	19.7
50.0	50.7	0.97	0.99	19.5
100.0	101.2	1.38	1.40	19.4
150.0	151.7	1.68	1.71	19.3
200.0	202.2	1.95	1.97	19.2
300.0	303.2	2.38	2.41	19.2
500.0	505.1	3.08	3.11	19.1

*Note:* The ‘actual’ values include the effect of imaging gradients and were used in the IVIM analysis. The numbers present an average over six diffusion directions. For velocity‐compensated encodings, the variability in all experimental parameters (nominal vs. actual) was negligible with differences in *m*_1_ at 0.001 s/mm across all b‐values, while for *b* the differences were negligible (1e^−7^ s/mm^2^).

**FIGURE 2:**
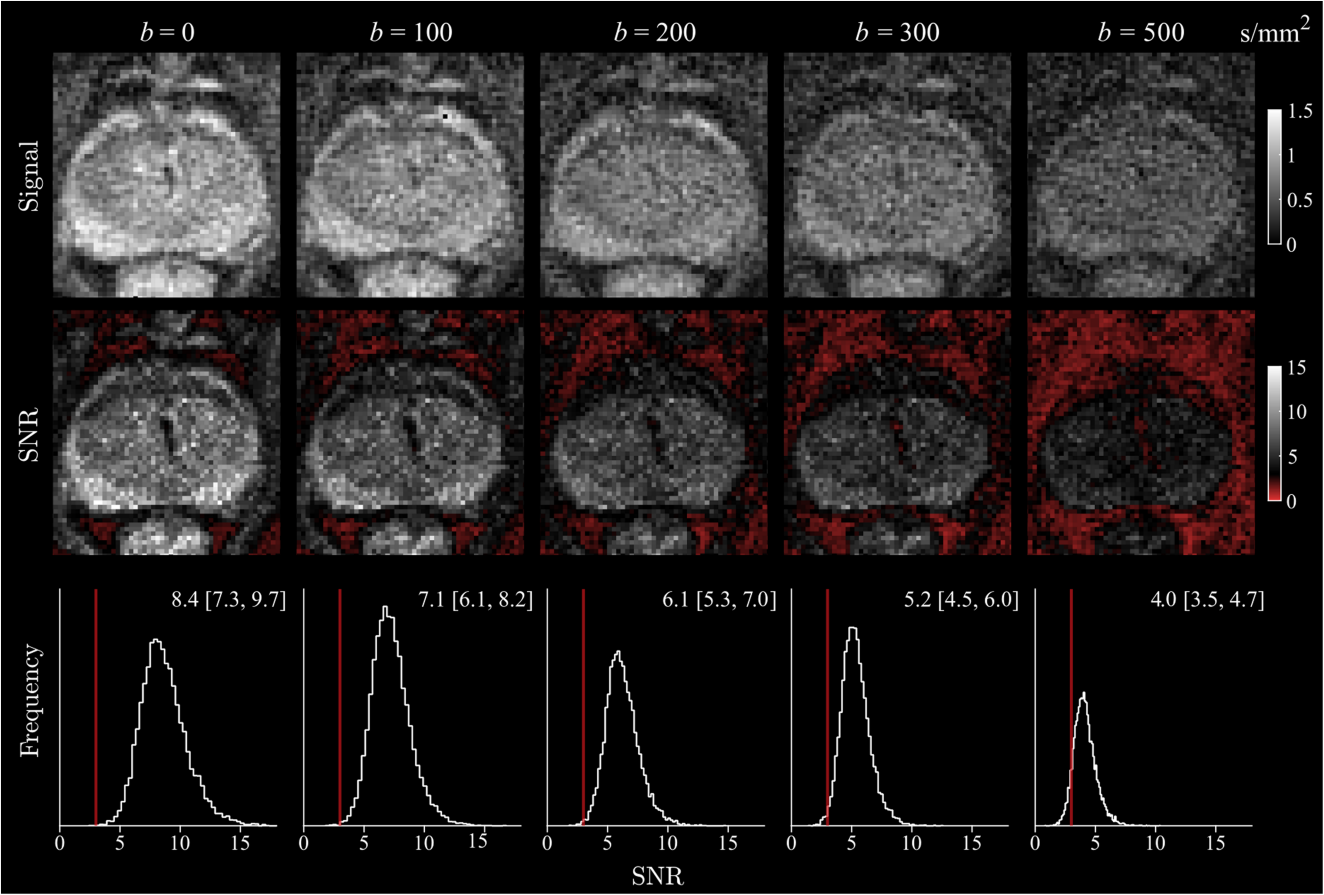
Single‐shot signals and SNR maps at selected *b*‐values for a representative subject (ID = 2; Table 1) in a single transverse slice (top) and SNR distributions across pulled data (scan and rescan together) from all five included subjects (bottom) with median and interquartile range. The example voxel‐wise SNR maps from scan 1 are colour‐coded in red–white, where red voxels indicate SNR < 3. Generally, prostate tissue exhibited SNR > 3, with little number of voxels at the highest b‐values where SNR < 3 was observed. The red vertical line marks SNR = 3.

The voxel‐wise SNR for a single patient (ID = 2) is shown in Figure 2. The SNR of high in‐plane spatial resolution data was mostly higher than 3 within the prostatic gland. Pooled SNR analysis across subjects revealed that at b‐value of 500 s/mm^2^, 91% of voxels had SNR > 3, while for all remaining b‐values, ≤1% of voxels were classified as low SNR.

**FIGURE 3:**
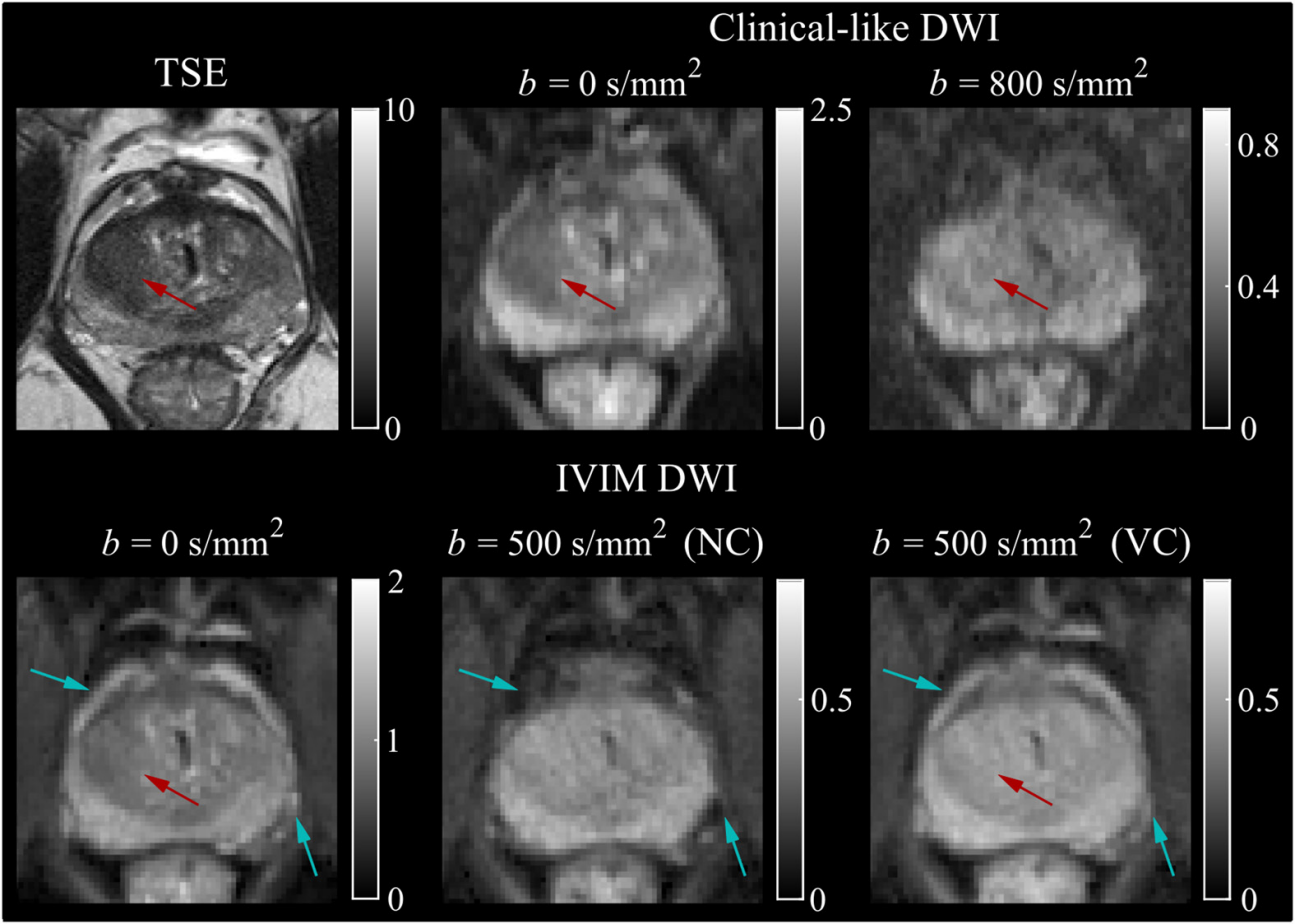
Examples of a standard ‘clinical‐like’ diffusion‐weighted imaging (DWI) protocol (top row) and the proposed IVIM DWI protocol (bottom row; NC—non–velocity‐compensated, VC—velocity‐compensated) from patient ID = 2. All images were averaged over repetitions (b‐values and directions). The geometric consistency of fine anatomical features was well preserved in IVIM data, while ‘clinical‐like’ data had relatively low spatial resolution and more prominent artifacts due to eddy currents and local field inhomogeneity. The red arrow points to a cancerous lesion in the central zone, and blue arrows point to the venous plexus (anterior) and neurovascular bundle (posterior) around the prostate gland, which were most visible when using velocity‐compensated encoding.

Diffusion‐weighted data obtained using proposed imaging framework had a higher nominal spatial resolution than the one obtained with conventional ‘clinical‐like’ protocol and better structural fidelity (e.g., visually better defined tissue edges) when compared to the T_2_‐weighted scan (Figure 3). We found that the venous plexus and the neurovascular bundle, which are in close proximity to the prostate gland, could be easily distinguished by the contrast between images acquired with velocity‐compensated and non‐compensated diffusion encoding, respectively (Figure 3, cyan arrows).

**FIGURE 4:**
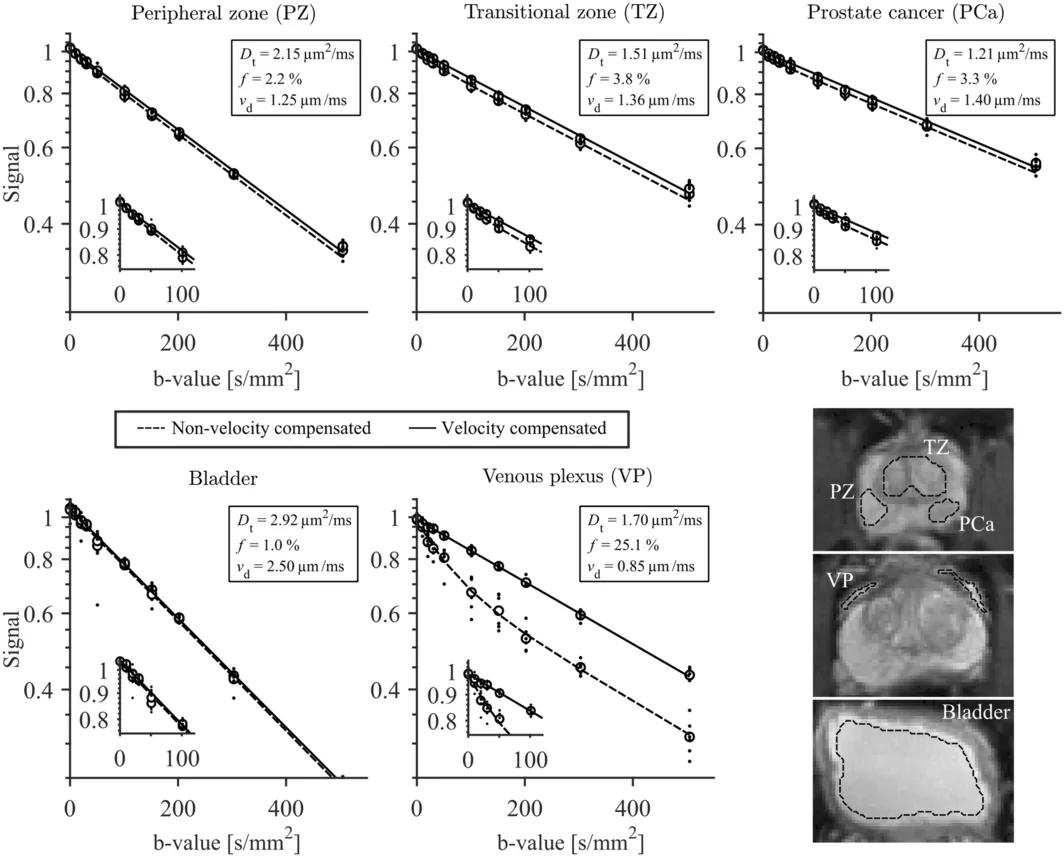
Signal vs. b‐value and flow encoding with estimated parameters (*D*_t_—tissue diffusivity, *f*_b_—perfusion fraction, *v*_d_—velocity dispersion) from a representative subject. The signal dynamics are presented for transitional (TZ) and peripheral zones (PZ), cancerous tissue (PCa), venous plexus (VP) and bladder. The insets show the signal at low b‐values. Manually drawn ROIs are depicted on *b* = 0 s/mm^2^ images. The non‐zero perfusion fraction in bladder was likely due to noise. The plot displays the data with lines representing the fit for the velocity‐compensated and non–velocity‐compensated encoding schemes, dots showing the signal obtained for each rotation, and circular markers indicating the average signal at each b‐value. Note that the reported fits were estimated using ROI‐averaged signals (the mean overall directions per b‐value and per waveform).

For a selected patient (ID = 1), VC‐NC signal dynamics and corresponding IVIM fit in different tissue classes are shown in Figure 4. With velocity‐compensated encoding, the signals resulted in approximately mono‐exponential attenuations for the range of sampled b‐values. The scale of separation between the VC and NC signals was observed to vary depending on tissue type. Specifically, for the selected patient, in cancer and peripheral and transitional zones, the split between the two encodings is much smaller yielding perfusion fractions within 2.2%–3.8%. For structures surrounding the prostate, e.g., neurovascular bundle, a marked separation of VC and NC signals is observed, for which the estimated perfusion fraction is 25.1.

**FIGURE 5:**
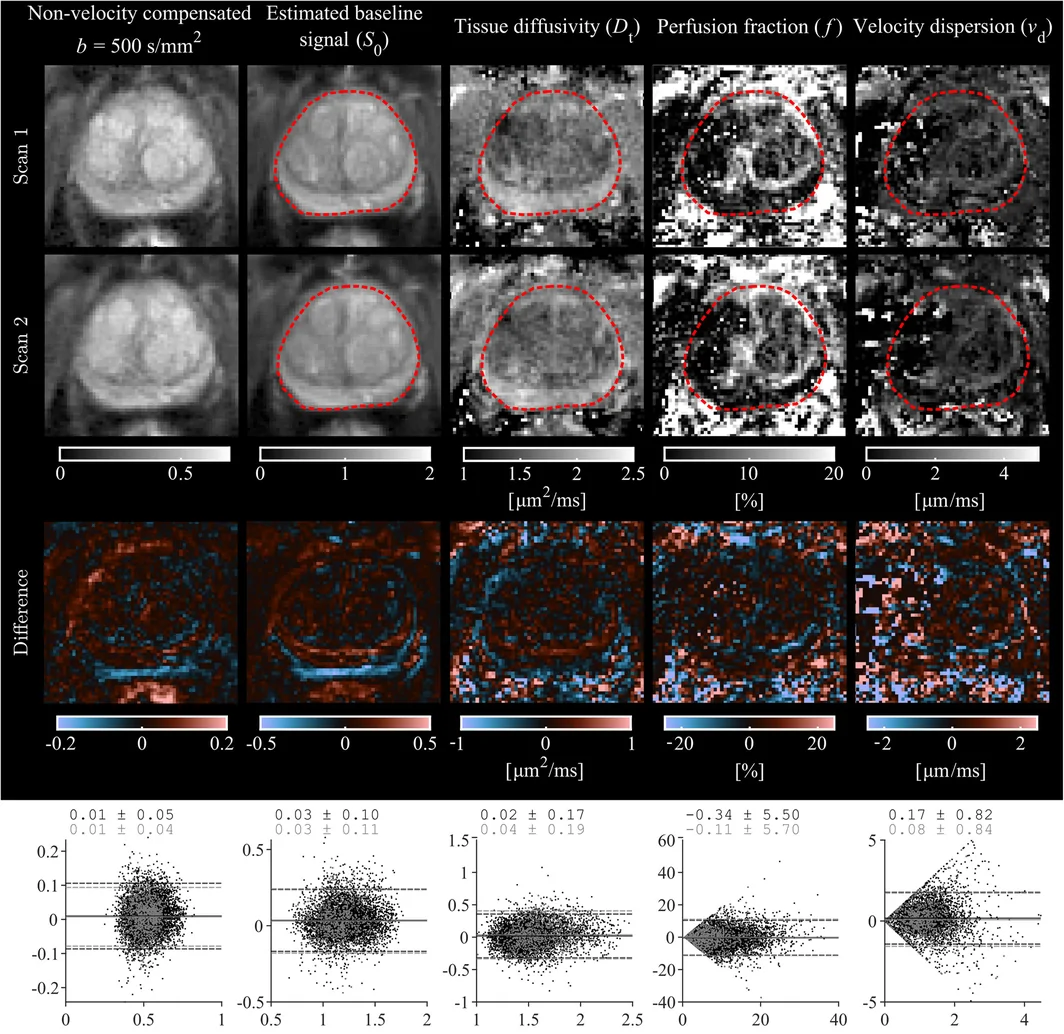
Signal and IVIM parameter maps from repeated acquisitions and analysis of repeatability for one case subject from the study. For easier visual assessment of parametric maps, a prostate outline was added (red dashed outline). The voxel‐wise difference between acquisitions (scan 1 and scan 2) is colour‐coded in blue–black–red. The difference maps show the largest differences at gland borders where small misalignment between the first and second acquisition causes larger parameter discrepancy. Bland–Altman plots show the distributions of voxel‐wise differences of parameters in the depicted slice (grey) and the whole gland (black). Corresponding colour coding is used to show horizontal lines and numerical values for the average and the limits of agreement (average ± 1.96 standard deviations). The analysis shows that the biases are negligible compared to parameter variance. Note that the estimated precision pertains to the per‐voxel parameter uncertainty; analysing the average over multiple voxels is expected to markedly improve the precision.

The test–retest voxel‐by‐voxel analysis of IVIM parametric maps and measured NC signals for a selected patient (ID = 3) is presented in Figure 5. The parameter maps of both acquisitions are qualitatively similar; the differences are shown in transversal slices and visualised as Bland–Altman plots. Overall, the analysis showed that the biases are negligible compared to parameter variances. Notably, the highest repeatability was observed for the NC signal, estimated baseline signal and tissue diffusivity, while the precision of perfusion fraction was the lowest.

For the selected patient (ID = 3) in Figure 5, tissue diffusivity maps differed in regions, with higher values observed in the peripheral zones in comparison to the transitional zone. The perfusion fraction maps exhibited spatial patterns that could be visually related to non‐diffusion weighted images. Higher perfusion fraction values were observed in regions of transitional zones, right next to the transition area between prostate zones or around benign prostatic hyperplasia nodules (Figure 5, right transitional zone). By contrast, lower perfusion fraction values were observed in some of the benign prostatic hyperplasia nodules (left transitional zone). The maps of velocity dispersion were spatially uniform, with higher values wherever perfusion fraction values were higher.

**FIGURE 6:**
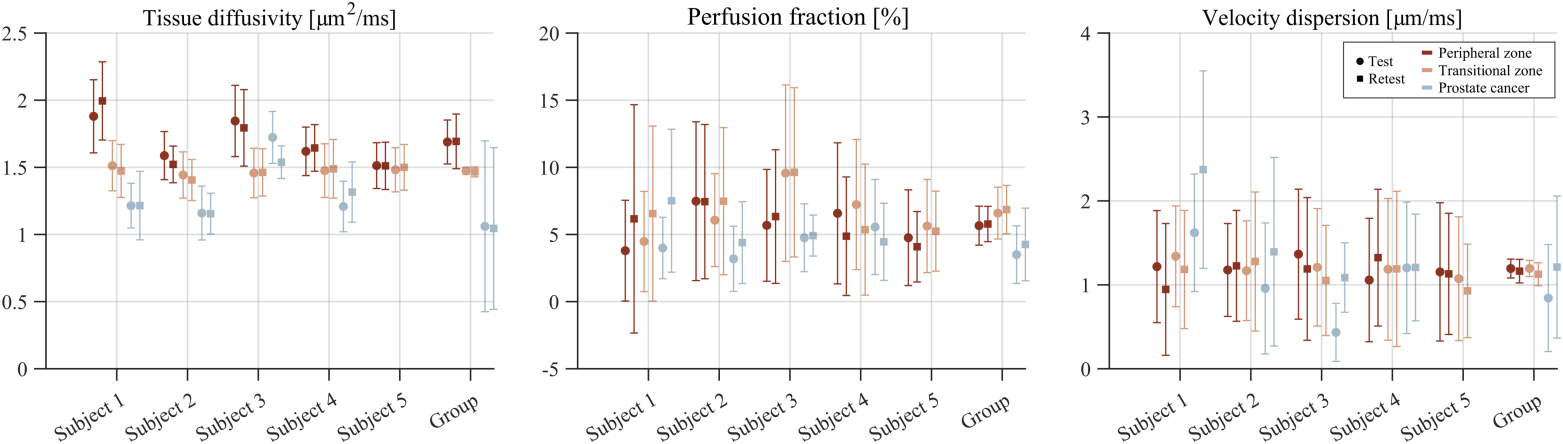
Mean and standard deviation of IVIM parameters across subjects and as a group for test and retest session for three tissue classes. The best repeatability within subject and at group level was observed for tissue diffusivity, followed by perfusion fraction. As expected from the literature, the highest tissue diffusivity was reported for peripheral zone and the lowest for prostate cancer lesions. For subject 3, transitional zone showed the lowest diffusivity, likely to be an effect of abundance of BPH nodules. At group level, the highest perfusion fraction was reported for transitional zone, and the lowest for prostate cancer lesions, despite high variability across subjects. Due to high variability of velocity dispersion within subjects, careful consideration and further validation are required. Note that subject 5 is an age‐matched healthy subject (no confirmed prostate lesion).

In the ROI‐based analysis (Figure 6), repeatability on patient and group levels was the highest for tissue diffusivity, followed by the perfusion fraction. The highest tissue diffusivity was reported for peripheral zone and the lowest for cancerous lesions. For patient ID = 3, the transitional zone showed the lowest diffusivity, likely to be an effect of abundance of benign prostatic hyperplasia nodules. At the group level, the highest perfusion fraction was reported for the transitional zone, and the lowest for cancerous lesions, despite the high heterogeneity of parameter values across subjects. The velocity dispersion parameter was repeatable in healthy‐appearing peripheral and transitional zones at subject and group level, while in cancerous lesions, the differences in test–retest parameters were non‐negligible.

## 4 Discussion

We have demonstrated the technical feasibility and performance of IVIM with velocity‐compensated encoding at high gradient amplitudes and spiral readouts. This combination of methods yielded unprecedented signal and IVIM parameter quality that could be robustly acquired in a cohort of prostate cancer patients. To our knowledge, this is the first study of velocity‐compensated IVIM in prostate imaging. Our research therefore provides initial evidence that the velocity‐encoding affects signal characteristics in and around the prostate, which may serve as a baseline in the design of future experiments for IVIM imaging.

The proposed framework yielded IVIM data of high in‐plane spatial resolution with clearly improved structural fidelity (Figure 3). Combined techniques contributed to superior signal precision, which is an underlying factor for improvements in the IVIM fitting (Figures 4 and 5). Interestingly, velocity‐compensated encoding recovered signal in fine anatomical structures around prostate (venous plexus and neurovascular bundles), which could be relevant to nerve‐sparing radical prostatectomy [53].

The estimated diffusion parameters fall within the range of expected values, e.g., tissue diffusivity is the highest in peripheral zone, followed by transitional zone and prostate cancer lesions. The reported perfusion fraction is lower than what was thus far reported in the literature, e.g., by He et al. (10% for non‐cancerous tissue) [5] or Yao et al. (9.6% for cancerous regions) [54]. Prior research indicates that the perfusion fraction is a function of TE, such that a shorter TE yields a lower estimated perfusion fraction value [55]. Since this is the first, and small scale, study of velocity compensation and strong gradients in prostate cancer, we emphasise that more extensive studies of clinical feasibility are warranted to establish quantitative relationships between the measured VC‐NC IVIM parameters and underlying prostate tissue microstructure.

The use of variable velocity‐compensation was motivated by prior works showing the improvement of precision and accuracy of estimated IVIM parameters [17], as compared to conventional diffusion encoding. Previously, the quality of estimated parameters has been aided by explicit or implicit regularisation [17, 56]. A convenient approach is segmented fitting, wherein the diffusivity is first estimated from signal at moderate b‐values (200–1000 s/mm^2^), whereafter it is fixed to allow a subsequent estimation of the perfusion fraction [57]. Although this improves the precision of the estimated parameters, it assumes mono‐exponential signal decay at moderate b‐values and no signal contribution from blood. Violations of these assumptions lead to biased parameter estimates. In the present study, we deliberately chose a simple NLLS to highlight the underlying data quality. More sophisticated estimation methods are expected to yield even better results, but would risk conflating two sources of improvement, i.e., data quality and fitting strategy. NLLS fitting remains the field's reference standard and provides a meaningful benchmark against which future fitting strategies can be compared [16]. The high‐quality data afforded by our framework may constitute an ideal substrate for training or validating alternative fitting procedures [58] and we view this as a natural and compelling direction for future work.

We acknowledge that the fitting method used herein still required that we fix the blood diffusivity (*D*_b_) to a constant value (*D*_b_ = 1.54 μm^2^/ms). While this practice is widely adopted, imposing an inaccurate *D*_b_ could cause inaccurate estimation. Findings by Funck et al. [45] suggest that the true *D*_b_ depends on the haematocrit level of the blood, which is subject‐specific. Using their simulation framework [45], simulations at shorter encoding times and TEs showed no dependence on the encoding time and no clear dependence on TE. In lieu of an accurate per‐subject estimation of *D*_b_, this error was accepted.

The main contribution to the quality of the diffusion‐weighted images was the spiral readout [26]. Currently, a condition for the implementation of spiral readout, with its custom image reconstruction and field monitoring, requires both specialised field monitoring hardware and expert personnel. Although this presents a clear challenge to clinical translation, several improvements and alternatives are expected to reduce the barrier to entry. For example, alternative correction techniques, such as gradient impulse response function, can provide more accurate k‐space encoding trajectories [59].

Throughout, the implementation of this study was facilitated by the ultra‐strong gradients. The benefit of strong gradients is manifested in the relatively short duration of the diffusion encoding and achievable higher b‐values per unit time. This is especially relevant for velocity‐compensated gradient waveforms due to their reduced efficiency [60]. Specifically, using bipolar gradients with similar duration to those presented by Ahlgren et al. [17] at 80 mT/m, we achieved more than twice their maximal b‐value. Moreover, short encoding times also increase the probability that capillary flow is in the ballistic regime, emphasising the signal difference that is attributed to the perfusion fraction [61]. While a dedicated assessment of diffusion encoding times [18] as well as tissue relaxation characteristics [55] were outside the scope of this work, further investigations are warranted to assess their influence on parameter estimation.

Prior investigations have demonstrated limited diagnostic value of in vivo IVIM in prostate cancer imaging [5], which could be attributable to poor image quality. In this study, we investigated repeatability of velocity‐compensated IVIM and its parameters at the subject and group level. At subject level, the acquired high b‐value signals, estimated baseline signal and tissue diffusivity showed negligible bias and high precision. By contrast, the repeatability was lower for the perfusion fraction and velocity dispersion. The voxel‐wise differences for these two parameters exhibited relatively large variance and were truncated by the fitting bounds at low parameter values. We emphasise that the voxel‐wise analysis is maximally sensitive to errors caused by noise and geometric inconsistency or shifts of the object. A similar ROI‐based analysis exhibited both smaller variance and smaller errors as seen by the high repeatability of average parameter values within given tissue class within subjects and at group level (Figure 6).

We acknowledge several limitations of the present study. Although the included MRI system affords great encoding capabilities, current results are based on five prostate cancer patients, a single experimental design and analysis method, as well as a non‐standard post‐processing and reconstruction pipeline. While the presented results demonstrate the value of our approach, the translation to conventional MRI systems remains as a future challenge. Nevertheless, velocity‐compensated diffusion encoding is compatible with conventional MRI hardware and is relatively straightforward to implement [62, 63]. The primary drawback is that velocity compensation reduces the maximal b‐value for a given sequence setup [64], which necessitates longer echo times. In a preliminary study, we have demonstrated VC‐NC IVIM on a clinical MRI system with 200 mT/m gradients with encoding times of 36 ms at *b* = 500 s/mm^2^ and EPI readout, which yielded an echo time of 70 ms [65]. At 80 mT/m and *b* = 500 s/mm^2^, velocity compensation requires an extension of the echo time by 10–20 ms, assuming asymmetric gradient waveforms [63]. Finally, it remains the topic of active research to map the value of various levels of motion compensation across analysis methods and organs [16]. In prostate cancer, we expect that a larger clinical cohort with a wider range of tumour grades is necessary to establish the value of velocity compensation for IVIM‐derived biomarkers. Indeed, velocity‐compensated IVIM is still relatively nascent and further research is needed to arrive at a consensus regarding b‐ and m_1_‐value sampling, protocol setup, image preprocessing and choice of fitting algorithm are all open questions for velocity‐compensated prostate IVIM.

## 5 Conclusions

By combining advanced experimental design with modern MRI hardware, we achieved unprecedented prostate IVIM data quality. Strong gradients and spiral readout enabled sampling at short diffusion and echo times, yielding high SNR. Velocity‐compensated encoding substantially affected IVIM dynamics that enabled improved parameter estimation. Together, our imaging and analysis framework demonstrated reliable IVIM in prostate cancer. This verification of technical feasibility may pave the way for further research on prostate IVIM with robust measurements.

## Author Contributions

**Malwina Molendowska:** conceptualisation, software, formal analysis, investigation, resources, writing – original draft, writing – review and editing, visualisation, funding acquisition. **Ivan A. Rashid:** conceptualisation, software, formal analysis, resources, writing – original and draft, writing – review and editing. **Kieran Foley:** formal analysis, investigation, resources, writing – review and editing, funding acquisition. **Lars Mueller:** software, methodology, writing – review and editing. **Lars E. Olsson:** supervision, writing – review and editing, funding acquisition. **Patrik Brynolfsson:** resources, software, supervision, writing – review and editing. **Filip Szczepankiewicz:** resources, project administration, supervision, writing – original and draft, writing – review and editing, funding acquisition.

## Conflicts of Interest

Patrik Brynolfsson is a shareholder of Hero Imaging AB and developer of the Hero platform.

## Supporting information


**Figure S1:** IVIM parameter maps from analysis using single‐step NLLS, median of 100 single‐step NLLS fits and two‐step NLLS fit. Parameter maps of tissue diffusivity were visually comparable across all three fitting approaches. Perfusion fraction and velocity dispersion maps from single‐step NLLS exhibited randomly distributed outliers, which were effectively eliminated when taking the median across 100 iterations or performing two‐step NLLS fit using local neighbourhood information.
**Figure S2:** Bland–Altman analysis for three IVIM parameters comparing the two‐step NLLS fit (red) and single‐step fit (grey); please note that the reference approach for comparisons is the median over 100 single‐step NLLS fits. Corresponding colour‐coding is used to present numerical values for the average and the limits of agreement. Across all three parameters, the two‐step fit shows consistently smaller bias and substantially narrower limits of agreement than the single‐step fit, indicating more precise and reproducible parameter estimation. The single‐step fit exhibits wider scatter and more outliers, particularly at higher perfusion fraction and lower velocity dispersion values, suggesting greater estimation instability under this approach. Points—difference of parameters; solid line—bias; dashed lines—limits of agreement.

## Data Availability

Due to ethical concerns, supporting data cannot be made openly available. Data can be provided upon request and signature of data sharing agreement. Please contact cubric@cardiff.ac.uk or malwina.molendowska@med.lu.se in the first instance.
