# Supplementary material for: Technical Feasibility of IVIM for Prostate Cancer Imaging Using High‐Amplitude Whole‐Body Gradient System With Spiral Readout and Velocity‐Compensated Diffusion Encoding
Source: NMR Biomed. 2026 Sep 9;39(10):e70384. doi: 10.1002/nbm.70384 (PMC13559045; doi:10.1002/nbm.70384)
Supplement: Supplementary file 1 [file NBM-39-e70384-s001.docx]

**Supplementary Materials for “Technical feasibility of IVIM for prostate cancer imaging using high-amplitude whole-body gradient system with spiral readout and velocity-compensated diffusion encoding”**

Malwina Molendowska^1,2*^, Ivan A Rashid^3^, Kieran Foley^4^, Lars Mueller^5^, Lars E Olsson^3,6^, Patrik Brynolfsson^6^, Filip Szczepankiewicz^1^

# *Analysis of precision and accuracy of IVIM fitting*

A known limitation of non-linear least-squares (NLLS) fitting is the dependence on initial parameter guesses — if poorly chosen, the optimizer may converge to a local rather than global minimum. One common strategy to mitigate this is to randomise the initial guess within predefined bounds and repeat the fit multiple times, retaining the best result. In our work, we adopted a two-step approach: an initial fit with a random guess, followed by a second fit initialised using the median of estimates in the local neighbourhood (3 × 3 pixels). To validate the accuracy and precision of this strategy, we compared it against single-step fits performed multiple times with randomised initialisation.

We conducted 100 independent iterations of a single-step fitting procedure in a single subject (whole prostate), each with random initial guesses drawn uniformly within the prescribed bounds. The resulting parameter distributions were summarised by their medians and compared against estimates from both a single execution of the single-step NLLS and the two-step fitting procedure described in the main manuscript. Agreement between methods with respect to reference –median over 100 single-step NLLS iterations— was assessed using Bland-Altman plots, with the mean as the bias measure and 2.5th and 97.5th percentile-based limits of agreement.

The parametric maps (tissue diffusivity, perfusion fraction and velocity dispersion) for single iteration of single-step NLLS, median over 100 single-step NLLS and two-step NLLS fitting are shown in Figure S1. All three fitting options produced visually comparable tissue diffusivity; however, the remaining parameter maps –perfusion fraction and velocity dispersion— exhibited randomly distributed outliers in the case of single-step NLLS, highlighting the instability of single-step fitting attributable to random initialisation. Importantly, evaluating a median over 100 single-step NLLS fits or performing two-step NLLS fit effectively eliminated these outliers, confirming that the instability arises from initialisation rather than the fitting procedure itself. can reproduce the results of 100 single-step fits. Furthermore, the two-step fitting procedure yielded results consistent with those obtained from 100 single-step iterations.

For all three parameters, Bland-Altman analysis demonstrated closer agreement for the two-step fit compared to the single-step fit with respect to the reference approach (Figure S2). For tissue diffusivity, the two-step fit showed a bias of 0.002 ± 0.005 μm²/ms, compared to 0.007 ± 0.048 μm²/ms for the single-step fit. Similarly, for perfusion fraction, bias was 0.13 ± 0.39% for the two-step fit versus 1.02 ± 5.31% for the single-step fit, and for velocity dispersion, bias was −0.06 ± 0.26 μm/ms versus −0.04 ± 0.74 μm/ms, respectively. Across all parameters, the two-step fit yielded consistently narrower limits of agreement than the single-step fit, indicating greater precision and reproducibility. The single-step fit showed wider scatter and more outliers, particularly at higher perfusion fraction and lower velocity dispersion values, suggesting reduced estimation stability.

Figure S1. IVIM parameter maps from analysis using single-step NLLS, median of 100 single-step NLLS fits and two-step NLLS fit. Parameter maps of tissue diffusivity were visually comparable across all three fitting approaches. Perfusion fraction and velocity dispersion maps from single-step NLLS exhibited randomly distributed outliers, which were effectively eliminated when taking the median across 100 iterations or performing two-step NLLS fit using local neighbourhood information.

Figure S2. Bland-Altman analysis for three IVIM parameters comparing the two-step NLLS fit (red) and single-step fit (gray); please note that the reference approach for comparisons is the median over 100 single-step NLLS fits. Corresponding colour coding is used to present numerical values for the average and the limits of agreement. Across all three parameters, the two-step fit shows consistently smaller bias and substantially narrower limits of agreement than the single-step fit, indicating more precise and reproducible parameter estimation. The single-step fit exhibits wider scatter and more outliers, particularly at higher perfusion fraction and lower velocity dispersion values, suggesting greater estimation instability under this approach. Points – difference of parameters, solid line – bias, dashed lines – limits of agreement.
